# The Destiny of Articles When Pairing “Traditional”—With Open Access Sibling Journals

**DOI:** 10.1097/TP.0000000000004293

**Published:** 2022-09-19

**Authors:** Tamar A.J. van den Berg, Stan Benjamens, Stephan J.L. Bakker, Jeremy R. Chapman, Edward K. Geissler, Robert A. Pol

**Affiliations:** 1 Department of Surgery, University of Groningen, University Medical Center Groningen, Groningen, The Netherlands.; 2 Department of Surgery, Maasstad Hospital, Rotterdam, The Netherlands.; 3 Department of Internal Medicine—Nephrology, University of Groningen, University Medical Center Groningen, Groningen, The Netherlands.; 4 Centre for Transplant and Renal Research, Westmead Institute for Medical Research, The University of Sydney, Sydney, NSW, Australia.; 5 Department of Surgery, Experimental Surgery Section, University Hospital Regensburg, Regensburg, Germany.

## Abstract

**Figure:**
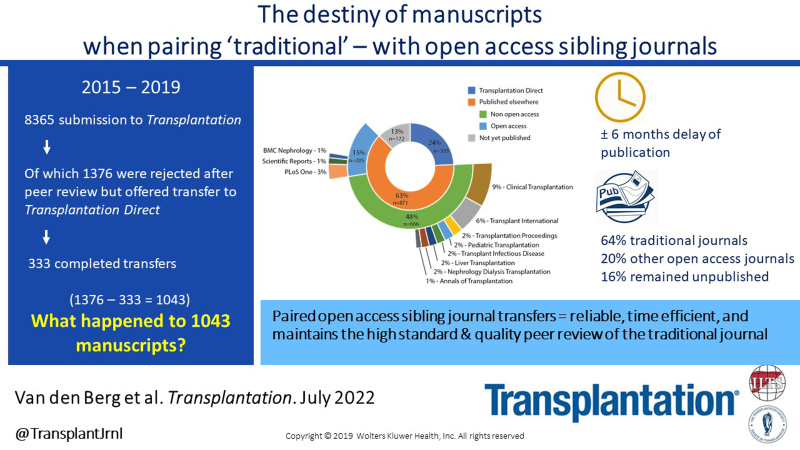

Open access publishing provides a model in which authors pay the costs of publication. In the “traditional” approach, in contrast, costs are mostly covered by the reader.^1–3^ It is available both through traditional journals and specific open access journals, which have been established by sponsoring professional associations. *The Transplantation Society* and Wolters Kluwer, the publisher of *Transplantation (TPA*), established *Transplantation Direct* (*TXD*) as an online open access journal with specific goals and a continuous publishing mode, commencing publication in February 2015.^4^ Articles may be submitted directly to *TXD* or may be transferred after initial submission to *TPA*. The editors of *TPA* have the option to offer authors transfer of their articles to *TXD* if it is assessed to be of high quality, meeting specific aims and quality thresholds of *TXD* while not being deemed of sufficient priority for publication in *TPA*. Authors may respond that they “Agree to Transfer” or “Decline Transfer.” We conducted an analysis of 5 y of this practice and are thus able to provide insight into the outcome of such journal pairing by examining the destination of articles, which are offered the opportunity to flow from a traditional journal to an online open access paired journal.

All articles rejected by *TPA* after peer-review and offered transfer to *TXD* between 2015 and the end of 2019 were included in this bibliometric analysis. Data were derived from the submission system operated by the editorial offices of *TPA* and *TXD* and included the date of final decision, title and authors of the article, editorial article status if authors were offered transfer, and whether the transfer was received by *TXD*. All articles not published in *TXD* were sought on PubMed and Google Scholar by 2 independent reviewers (T.B. and S.B.) using key words from the title and first author names. Destination journals were categorized as either full open access or traditional, using the Directory of Open Access Journals,^5^ and the 5-y impact factor (IF), calculated from the years 2015 to 2019, was derived from the Journal Citation Reports provided by the Web of Science Clarivate Analytics Index System.^6^ The study was registered at the University Medical Center Groningen Research Register (no. 202100708). Data were accessed as an editorial system quality assurance study approved by the Executive Editorial Committee of Transplantation. All data retrieved remain confidential, no journal authorships are disclosed beyond the 2 independent reviewers, and all identifying data have been eliminated after articles were accepted for publication.

*TPA* received 8365 article submissions between 2015 and 2019, of which 1969 (23.5%) were published, 1170 (14.0%) were rejected after editorial “desk” review, and 5226 (62.5%) were rejected after full peer review (Figure 1). Of the latter group, 1376 (26.3%) articles were offered transfer to *TXD*. Authors accepted transfer of 451 articles, but in the end, only 333 successfully went through the review process and were published in *TXD*. Of the remaining 118, the article was not revised satisfactorily (n = 11), author declined to revise (n = 9), submission was removed by the author (n = 37), submission was removed by the editor (n = 3), submission was removed from the system because of prolonged inactivity (n = 52), and a small number was still being revised at the time of the analysis (n = 6). Thus, a total of 1043 articles offered transfer were not published in *TXD*. These articles were subjected to a systematic literature search. It appeared that 871 (83.5%) of these articles were published elsewhere, of which 666 (76.5%) articles were published in traditional journals and 205 (23.5%) in open access journals (Figure 2). A total of 172 (16.5%) articles remained unpublished at the time of the search. Of the articles published elsewhere, the duration between the date of article rejection by *TPA* and the date of final publication was markedly shorter when authors opted for *TXD* than for publication in another journal (102 [67–171] versus 264 (177–393) d, *P* < 0.001). The number of transfer offers and subsequent transfers published in *TXD* did not change over the years, with 250 to 290 articles offered a transfer each year, of which 22% to 28% were actually transferred and published.

**FIGURE 1.**
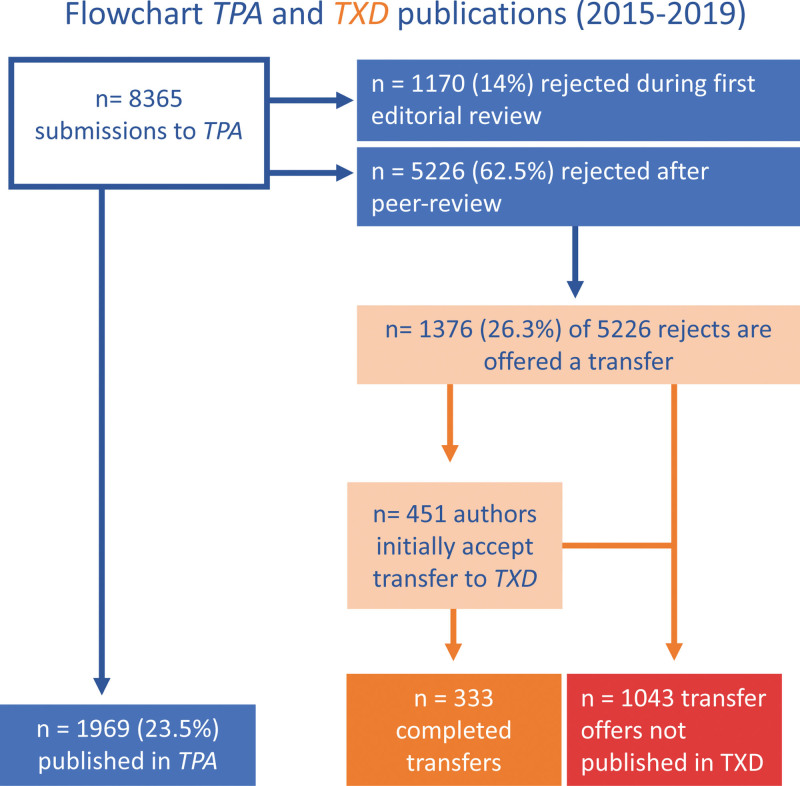
Flowchart *TPA* and *TXD* articles (2015–2019). *TPA, Transplantation*; *TXD, Transplantation Direct.*

**FIGURE 2.**
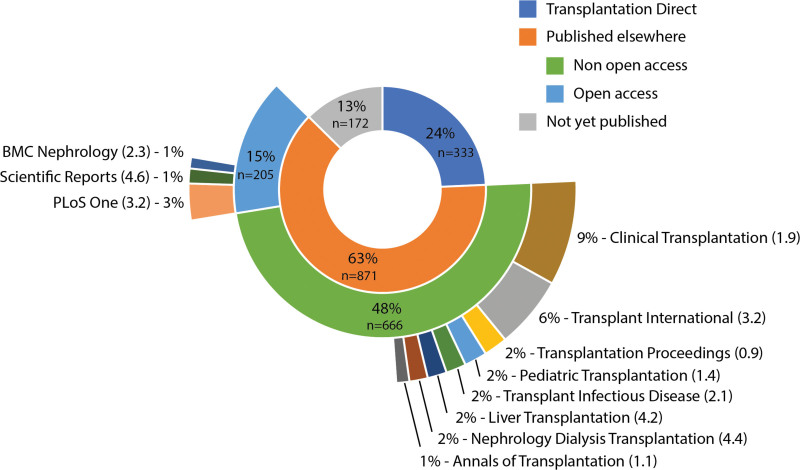
Sunburst chart of publication destination of articles offered transfer from *Transplantation* to *Transplantation Direct* (2015–2019, n = 1377). Five-year impact factor within parentheses. *Transplantation* 5-y impact factor (4.0).

This analysis demonstrates the choices made by authors as they seek to publish their article, as well as the tradeoffs of cost and time to publication that they experience as their articles flow to publication in both traditional and open access journals. About three quarters of authors whose articles were eventually published after declining transfer did so in conventional journals, and only a quarter published in an open access journal indexed by the Directory of Open Access Journals. Of critical relevance, this approach resulted in a substantially longer time to publication (mean additional time to publish of 181 d). The considerations that contributed to authors’ decisions are unknown. Publication in a paired open access journal through transfer is faster, more reliable and a more sustainable way to use peer-review, especially as it has become increasingly difficult to find suitable and willing expert peer reviewers. However, author publication costs are higher, and most open access journals have a lower IF. Although some universities and research institutions have established arrangements to meet article processing charges and publications costs, others, especially in emerging economies, do not, creating accessibility issues for these researchers.

This analysis has a number of limitations: we analyzed the publishing behavior of *Transplantation’s* editors and authors, which may not necessarily be applicable to other journals; we did not examine all articles rejected by *TPA* but focused on those that were offered transfer to *TXD*; *TXD* started in 2015 at the start of the analysis period and thus did not have an IF to help guide authors, although the use of the alternative system for which TXD has a reported and rising CiteScore (3.7)^7^ may assist.

Pairing traditional journals with open access sibling journals offers a reliable and time efficient opportunity for authors to publish their research while maintaining validation of the high standard and quality peer review of the traditional journal. This analysis provides an insight into editor and author decision making cascading between journals, especially with sibling journals offering a transfer option for articles of particular interest. Authors appear to opt for the highest IF journal that will accept their paper, and many opted for traditional journals perhaps to reduce publication costs. The downside of these decisions was the delay of publication by about 6 mo with many articles still being published with open access charges.

## ACKNOWLEDGMENTS

We would like to thank the editorial office of *Transplantation* and *Transplantation Direct* for assistance.

## Supplementary Material
