# Supplementary figures and images for: The Destiny of Articles When Pairing “Traditional”—With Open Access Sibling Journals
Source: Transplantation. 2022 Sep 19;107(2):300–2. doi: 10.1097/TP.0000000000004293 (PMC9875835; doi:10.1097/TP.0000000000004293)

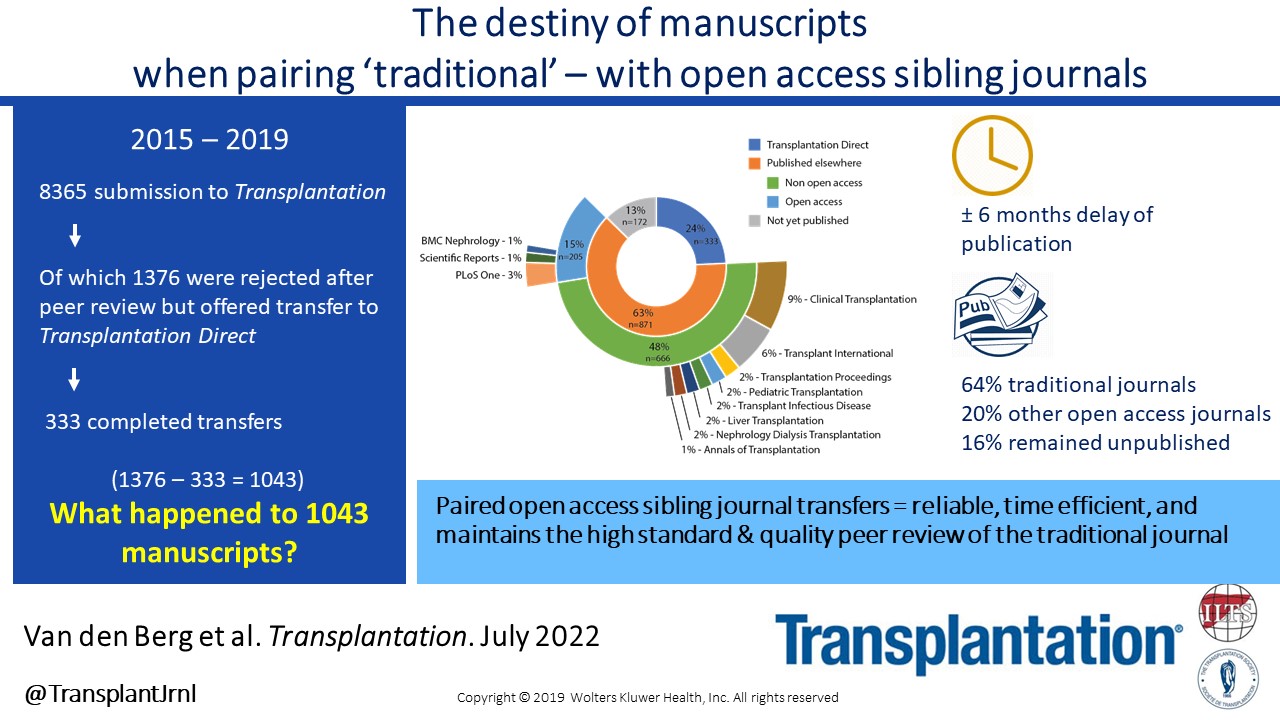

Supplement: Supplementary file 1 [file tp-107-300-s001.jpg]
